# Myocardial TGFβ2 Is Required for Atrioventricular Cushion Remodeling and Myocardial Development

**DOI:** 10.3390/jcdd8030026

**Published:** 2021-03-02

**Authors:** Aniket Bhattacharya, Nadia Al-Sammarraie, Mengistu G. Gebere, John Johnson, John F. Eberth, Mohamad Azhar

**Affiliations:** 1 Department of Cell Biology and Anatomy, University of South Carolina School of Medicine, Columbia, SC 29209, USA; Aniket.Bhattacharya@uscmed.sc.edu (A.B.); Nadia.Al-Sammarraie@uscmed.sc.edu (N.A.-S.); Mengistu.Gebere@uscmed.sc.edu (M.G.G.); John.Johnson@uscmed.sc.edu (J.J.); John.Eberth@uscmed.sc.edu (J.F.E.); 2 William Jennings Bryan Dorn VA Medical Center, Dorn Research Institute, Columbia, SC 29209, USA

**Keywords:** TGFβ2, myocardium, atrioventricular cushion, AVSD, mitral valve, SMAD2

## Abstract

Among the three transforming growth factor beta (TGFβ) ligands, TGFβ2 is essential for heart development and is produced by multiple cell types, including myocardium. Heterozygous mutations in *TGFB2* in patients of connective tissue disorders result in congenital heart defects and adult valve malformations, including mitral valve prolapse (MVP) with or without regurgitation. *Tgfb2* germline knockout fetuses exhibit multiple cardiac defects but the role of myocardial-TGFβ2 in heart development is yet to be elucidated. Here, myocardial *Tgfb2* conditional knockout (CKO) embryos were generated by crossing *Tgfb2*^flox^ mice with *Tgfb2*^+/−^; *cTnt*Cre mice. *Tgfb2*^flox/−^ embryos were normal, viable. Cell fate mapping was done using dual-fluorescent *mT/mG*^+/−^ mice. Cre-mediated *Tgfb2* deletion was assessed by genomic PCR. RNAscope in situ hybridization was used to detect the loss of myocardial *Tgfb2* expression. Histological, morphometric, immunohistochemical, and in situ hybridization analyses of CKOs and littermate controls at different stages of heart development (E12.5–E18.5) were used to determine the role of myocardium-derived TGFβ2 in atrioventricular (AV) cushion remodeling and myocardial development. CKOs exhibit a thin ventricular myocardium, AV cushion remodeling defects and developed incomplete AV septation defects. The loss of myocardial *Tgfb2* resulted in impaired cushion maturation and dysregulated cell death. Phosphorylated SMAD2, a surrogate for TGFβ signaling, was “paradoxically” increased in both AV cushion mesenchyme and ventricular myocardium in the CKOs. Our results indicate that TGFβ2 produced by cardiomyocytes acting as cells autonomously on myocardium and via paracrine signaling on AV cushions are required for heart development.

## 1. Introduction

Transforming growth factor beta (TGFβs) are a superfamily of profibrotic, anti-inflammatory, pleiotropic cytokines involved in diverse aspects of development and disease [1]. TGFβs are important players in initiating coronary circulation and play a cardioprotective role to prevent myofibrillar loss, extracellular matrix (ECM) degradation and cardiomyocyte apoptosis [2,3]. They are upregulated upon cardiac injuries (e.g., in infarcted myocardium) where they play an inflammatory role to contain the damage as well as initiate repair [4]. Among the three major ligands, TGFβ2 plays a crucial role in sculpting the developing heart. It is expressed by the myocardium, endocardium, epicardium and cushion mesenchyme cells during early heart development (E9.5–E11.5), and continues to be expressed in the myocardium, epicardium, and aortic wall throughout embryonic life (E12.5–E18.5) [5,6]. Heterozygous mutations in *TGFB2* results congenital heart defects and adult aortic valve malformation and mitral valve disease or mitral valve prolapse [7,8]. There is very little known about the origin of mitral valve disease, but recent findings indicate that clear genetic and developmental abnormalities of AV cushion remodeling underlie the pathogenesis of mitral valve disease [9,10]. *Tgfb2* regulates critical cellular events during heart development, including the formation and differentiation of epithelial-to-mesenchymal transition (EMT)-derived endocardial cushions, post-EMT valve remolding, outflow tract (OFT) septation and alignment, and the development of the aortic arch [11,12,13,14].

The myocardium conducts heart wave impulses in the vertebrate heart. It has been debated if developmentally, the compaction of the cardiomyocyte layers precedes trabeculation or vice versa, a phenomenon which also exhibits species specific variations [15,16]. In mice, the tubular heart at E8.0 has endocardium extending ‘sprouts’ into the multilayered myocardium, which touchdown, ingress laterally, extend apically and terminate by E14.5 [17]. The myocardium extends outwards during this process to accommodate the branching trabeculae [18]. The process represents a delicate communication between the endocardium and myocardium through the cardiac jelly and involves the extensive and dynamic remodeling of the extracellular matrix [17]. A battery of signaling pathways have been found to be involved including Notch, neuregulin, BMP and TGFβ which exert their effects through effectors like *Has2*, *Vcan* (ECM genes) and *Adamts1* (metalloproteinase). Compaction defects in cardiomyocytes leads to non-compaction cardiomyopathy, a rare congenital disorder in which myocardial fibers fail to compact, leading to deep intertrabecular recesses that continue to communicate with the endocardium [19].

In mice, the systemic loss of TGFβ2 leads to embryonic valvular defects such as dysmorphic and abnormally thickened mitral valves and myocardial defects [13,20]. TGFβ2 is myogenic and *Tgfb2^−/−^* exhibit spongy myocardium along with other compaction defects such as a thin outer layer of the ventricular myocardium as well as the impaired myocardialization of the atrioventricular (AV) cushions [21,22,23]. Despite such severe cardiac defects in TGFβ2 germline knockout fetuses, to date date, there are no studies done on the specific developmental role of myocardium-derived TGFβ2. To address this, we crossed *Tgfb2*^flox/flox^ mice [20] to *cTnt*Cre mice, which expresses Cre recombinase under rat cardiac troponin T promoter and effectively deletes the floxed allele by E10.5 [24]. Myocardial *Tgfb2* conditional knockout (CKO) embryos were generated through genetic intercrossing and heart development was studied between E12.5–E18.5.

## 2. Materials and Methods

### 2.1. Mouse Strains and Breeding Scheme

All animal breeding and experiments described herein were approved by the Institutional Animal Care and Use Committee of the University of South Carolina. Mice were bred and housed at the University of South Carolina Animal Research Facility at the School of Medicine. Myocardial *Tgfb2* CKO (*Tgfb2*^flox/−^; c*Tnt*Cre) mice were generated by intercrossing *Tgfb2*^flox/flox^, *Tgfb2*^+/−^, and c*Tnt*Cre mice [20,24,25]. First, Cre males were crossed to *Tgfb2*^+/−^ females; the F1 males from this were then crossed to either *Tgfb2*^flox/flox^ females (20) or *Tgfb2*^flox/flox^, *mTmG*^−/−^ females (for lineage tracing experiments) [26] (Figure S1). Timed pregnancies (TP) were set up and the noontime of positive vaginal plug was counted as embryonic day (E) 0.5.

### 2.2. Embryo Collection, Processing, Genotyping, Histology and Cell Lineage Tracing

Embryos were collected from timed pregnant dams after euthanizing them with isoflurane overdose, thoroughly washed in ice-cold 1× PBS and fixed in 4% PFA at 4 °C overnight to 48 h (depending on the embryonic stage). For lineage tracing experiments, fixed embryos were cryoprotected in 30% sucrose overnight at 4 °C, embedded in OCT and sectioned at 10 μm. Slides were washed in PBS and mounted in Vectashield antifade mountant (H-1800, Vector Labs, Burlingame, CA, USA). *mTmG* images were viewed and acquired using Zeiss fluorescence microscope and EVOS FL Auto Imaging System (ThermoFisher, Inc., Grand Island, NY, USA). For histology, fixed embryos were processed manually through graded ethanol, cleared in xylene and embedded in paraffin. Routine histological examination was performed using hematoxylin and eosin (H&E) staining on 7 μm paraffin sections as well as 10 μm frozen sections [20]. Sections were de-paraffinized, dehydrated with graded series of ethanol, stained in hematoxylin (Anatech, Battle Creek, MI, USA), rinsed in acid alcohol, blued in 0.1% NaHCO_3_, counter-stained with eosin (Anatech), rehydrated and cleared in xylene. Slides were photographed under brightfield optics on a Nikon Optiphot-2 (AxioCam MRc Camera, Carl Zeiss Microscopy, LLC, White Plains, NY, USA).

Genotyping was carried out with genomic DNA extracted from embryonic tail biopsies. *Tgfb2* germline KO and floxed alleles were genotyped using IMF9-IMR9 [25] and IMF65 (CACCTTTTACCTACAGATGAAGTTGC), IMR65 (CTTAAGACCACACTGTGAGATAATCC), IMR66 (CAACGGGTTCTTCTGTTAGTCC) primer pairs, respectively, as described previously [20]. c*Tnt*Cre transgene was genotyped using OIMR 1084–1085 primers (Jackson Lab) with denaturation: 95 °C/2 min; annealing and amplification 95 °C/30 s, 52 °C/1 min, 72 °C/30 s for 35×, 72 °C/5 min; 4 °C hold (24). *mTmG* allele was genotyped as described in Muzumdar et al. [26].

To check the efficiency of *Tgfb2*^flox^ allele deletion, punch biopsies were obtained from fixed myocardium and DNA was extracted using QIAamp DNA FFPE Tissue Kit (56404, Qiagen, Germantown, MD, USA) as per manufacturer’s protocol. Cre-mediated recombination was assessed by genomic PCR with IMF86 (AAGGCGCATAACGATACCAC) and IMR88 (ACTGATGGCGAGCTCAGACC) with denaturation: 94 °C/3 min; annealing and amplification 94 °C/30 s, 58 °C/30 s, 72 °C/45 s for 35×, 72 °C/5 min; 4 °C hold [20].

### 2.3. TUNEL Assay

Apoptosis was detected using a FragEL DNA Fragmentation Detection kit (QIA33, EMD Millipore, Burlington, MA, USA), as per the manufacturer’s recommendation. Briefly, sections were de-paraffinized in xylene and hydrated through graded alcohol, briefly rinsed in tris-buffered saline (TBS) and permeabilized in 20 µg/mL proteinase K for 20 min (10 min for cryosections). Endogenous peroxidases were blocked using 3% H_2_O_2_ in methanol for 5 min, equilibrated in the buffer and TdT labeling reaction mix and enzyme applied, incubated at 37 °C for 90 min in a humidified chamber. Reaction was terminated with a stop buffer, rinsed in 1× TBS, blocked, and incubated with conjugate for 30 min, rinsed in TBS and developed with DAB/ H_2_O_2_. Sections were counterstained with 1/10th diluted hematoxylin for 1 min, blued in 0.1% sodium bicarbonate for 30 s, dehydrated through graded alcohol and cleared in xylene, followed by mounting in Vectamount permanent mounting medium (H-5000, Vector labs).

### 2.4. Cell Proliferation

Cell proliferation was detected immunohistochemically with the nuclear marker for proliferating cells phospho Histone H3 (Cat# 9701S, Cell Signaling, Danvers, MA, USA) in AV cushion sections from E12.5–13.5 embryos. Heat-mediated antigen retrieval was performed using 1× antigen retrieval solution (Vector) for 10 min (microwave). Sections were incubated overnight at 4 °C with 1:200 diluted primary antibody, followed by biotinylated link (30 min), streptavidin–HRP (30 min) and detected with DAB. Nuclei were counterstained with hematoxylin (H-3404, Vector) for 2 min. Images were acquired from 4–6 random fields (20×) covering both the AV cushion and myocardium. At least 2500 nuclei were scored for each embryo (NIH Fiji: Nucleus area ≥200 sq. pixel, circularity 0.25–1).

### 2.5. Immunohistochemistry

Cryosections were hydrated with 2 changes in 1× PBS and 1 change in deionized water (5 min each). Heat-mediated antigen retrieval was performed by dipping the slides in a mildly boiling 1× citric acid buffer (catalog no. S1700; Agilent Dako, Santa Clara, CA, USA) for 10 min in a microwave, cooled to room temperature and rinsed in PBS. Endogenous peroxidases were blocked with freshly prepared 0.5% H_2_O_2_/methanol for 30 min, followed by non-specific epitope blocking with 5% goat serum/0.1% Tween/0.02% sodium azide in PBS for 20 min. Avidin and Biotin blocking was performed as per the manufacturer’s recommendation (Cat# SP-2001, Vector), followed by overnight incubation at 4 °C in anti-pSMAD2 (1:3000, Millipore), anti-Periostin (1:1000, Cat#ab14041, abcam, Cambridge, MA, USA), anti-cardiac α-actin (Clone: HHF35, catalog no. M0635) (Agilant Dako, Santa Clara, CA, USA) or anti-MF20 (1:50, DSHB, Iowa City, IA, USA) antibodies (20). Slides were then washed and incubated with an appropriate biotinylated secondary antibody (1:200) for 30 min, followed by Avidin–Biotin complex (Cat# PK-6100, Vectastain Elite ABC HRP kit) for 30 min, washed in PBS, and finally developed with DAB/H_2_O_2_. Nuclei were counterstained with hematoxylin and sections were dehydrated through graded ethanol series, cleared in xylene and mounted.

### 2.6. RNAscope In Situ Hybridization (ISH)

RNA in situ hybridization (ISH) was performed with 2.5 HD Detection kit (Brown) (Cat# 322310, Advanced Cell Diagnostics, Newark, CA, USA), as per the manufacturer’s protocol. E12.5–13.5 embryos were probed for *Tgfb2* mRNA (Mm-*Tgfb2*, Cat# 406181, Advanced Cell Diagnostics). DapB was used as a negative control (Cat# 310043). Heat mediated target retrieval was done for 15 min in a microwave and protease treatment for 30 min at 40 °C in a Hybrez oven. Slides were counter stained with one-tenth diluted hematoxylin for 1 min. Images were obtained at 40× from 4–5 random fields per section and analyzed with Fiji (NIH) using the method described in ACD Tech Note TS 46-003. At least 500 nuclei were scored for each animal (cut-off: size ≥ 500 sq. pixel, circularity 0.25–1). *Tgfb2* signal was measured using “weka segmentation” plugin (Fast Random Forest classifier) with a size ≥1 sq. pixel, circularity 0.25–1. Each probe cluster was counted only once, irrespective of the number of dots present. Three embryos were scored in each group.

### 2.7. Statistics

GraphPad Prism (San Diego, CA, USA) was used to perform all statistical analyses for the comparisons between two groups (CKO and control). Student’s *t* test or the Mann–Whitney (nonparametric test) (two-tailed, for two-group comparison) statistical tests were applied depending on the data type and distribution. *p*-values of less than 0.05 were considered significant. Error-bars represent the standard deviations (SD) within the groups.

## 3. Results

### 3.1. cTntCre Efficiently Deletes Tgfb2 in Early Cardiomyocytes

**Figure 1:**
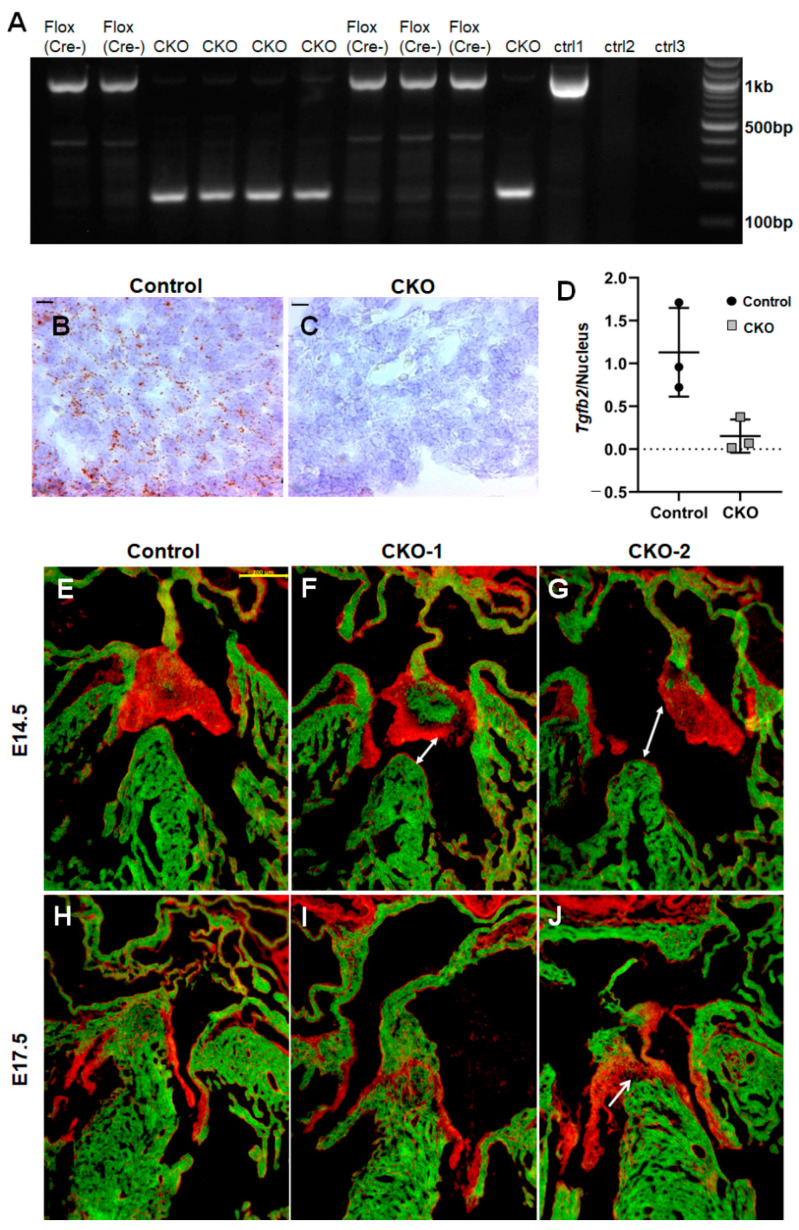
*cTnt*Cre efficiently deletes *Tgfb2* in the myocardium. (**A**) Genomic PCR with DNA extracted from the myocardium shows a near complete deletion of the *Tgfb2* floxed allele in the presence of Cre recombinase. All embryos containing the *cTnt*Cre transgene (CKO) contain the deleted 174bp band while their non-transgenic or wildtype (WT) littermates have the intact ~1 kb product amplification. DNA from tail biopsy (ctrl1) does not show the deleted band, suggesting Cre expression is specific to the myocardium (ctrl1). There is no amplification with wild-type DNA (ctrl2) as this PCR only detects floxed and deleted bands for *Tgfb2*. No DNA template control also indicated (ctrl3). (**B**–**D**) The expression of *Tgfb2* mRNA is significantly reduced in the ventricular myocardium of E12.5–13.5 *cTnt*Cre; *Tgfb2* conditional knock out (CKO) embryos compared to littermate controls (*p* = 0.0374, multiple *t*-test; *n* = 3). scale bar = 10 μm. (**E**–**J**) Cardiomyocytes were lineage traced using mT/mG dual fluorescent reporter where GFP expression acts as a surrogate for Cre activity. Some conditional knockouts (CKOs) show a greater myocardialization of AV cushion, marked by an increased number of GFP positive cells (**F**), compared to littermate controls (**E**). CKOs also have a range of cardiac malformations including dysmorphic AV cushion (**F**,**G**) and ventricular septal defect (VSD; **F**,**G**; white arrows) which may continue to persist over development (**J**) or be sealed (**I**). scale bar = 200 μm.

*cTnt*Cre is known to induce recombination as early as E7.5 in the early cardiomyocytes of a developing mouse heart and the floxed allele is effectively deleted by E10.5 (24). *Tgfb2* is also expressed in the precardiac mesoderm which gives rise to the tubular heart that subsequently folds, loops and septates to form the four-chambered heart. We restricted our study to the window of E12.5–18.5 to be able to observe the effect of myocardial TGFβ2 deficiency on mouse cardiogenesis (Figure 1 and Figure S1, Table 1). Myocardial *Tgfb2* CKOs were produced via a two-step breeding strategy (Materials and Methods, Figure S1) and littermate controls were used for comparison in every experiment. The CKOs did not exhibit any gross morphological defects, except for being slightly smaller than littermate controls (Figure S2).

To assess the extent of genetic recombination, we extracted DNA from myocardial punch biopsies from E17.5 embryos and PCR amplified both the *Tgfb2* conditional ready (i.e., floxed, Cre-negative) as well as the deleted allele (i.e., floxed, Cre-positive), as described in (20). A strong 174bp band for the deleted allele could be detected for all the animals that contained the *cTnt*Cre (also called (*TnnT2Cre*) transgene while it was absent in all non-transgenic control littermates (Figure 1A). To score the efficiency of deletion, we performed a densitometric analysis of the ~1kb floxed band that corresponds to the unrecombined DNA and found a ~30-fold enrichment in non-transgenic controls compared to *cTnt*Cre^Tg^, *Tgfb2*^+/f^ or *cTnt*Cre^Tg^, *Tgfb2*^−/f^ embryos. The residual amplication in the transgenic animals may be attributed to the presence of resident cardiac fibroblasts and endothelial cells in the myocardium which do not express *cTnt*Cre, along with the fact that no Cre recombinase is 100% percent effective. We did not detect the deleted *Tgfb2* allele in non-myocardial tissues of *cTnt*Cre^Tg^, *Tgfb2*^+/f^ embryos or observe any amplification with wild-type DNA, as expected (Figure 1A).

To examine the effect of the genomic deletion of *Tgfb2* on its expression levels, we carried out RNAscope in situ hybridization in E12.5–13.5 ventricular myocardium. Compared to littermate controls, CKOs exhibit a significant reduction in *Tgfb2* transcript expression (average number of puncta per nucleus) in the myocardium (*p* = 0.037, multiple *t* tests) corroborating the activity of *cTnt*Cre (Figure 1B–D). The residual signal may again be partly attributed to other, non-cardiomyocyte cells present in the myocardium which was further corroborated by the loss of *Tgfb2* only in the myocardium. Thus, the data indicate that *Tgfb2* expression is significantly downregulated in the myocardium of the CKOs compared to control fetal hearts.

**Figure 2:**
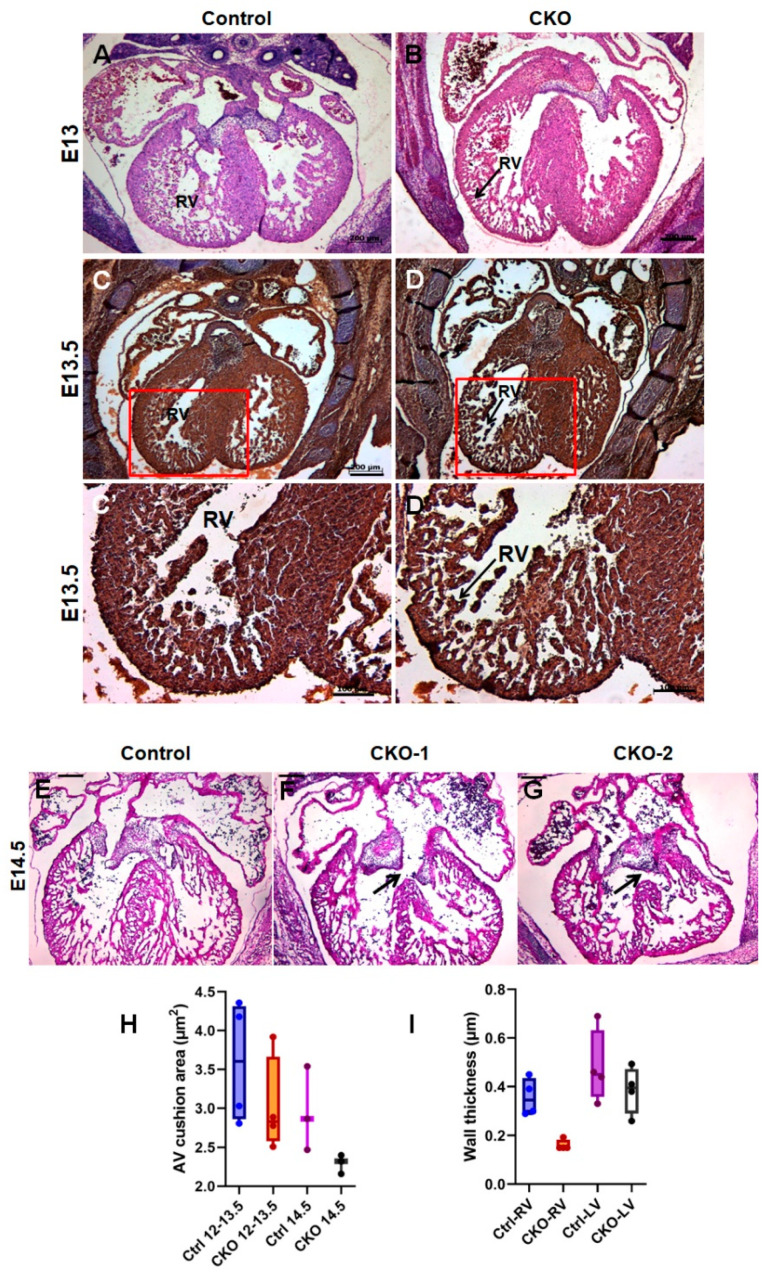
Conditional deletion of *Tgfb2* in early cardiomyocytes disrupts myocardial development, AV cushion remodeling, and AV septation. (**A**–**D**) Hematoxylin and eosin (H&E) (**A**,**B**) and cardiac muscle actin (**C**,**D**) staining show thinning of right ventricular myocardium in *cTnt*Cre; *Tgfb2*^−/flox^ (CKO) embryos compared to littermate controls (E13–E13.5). (**C′**,**D′**) are magnified views of the regions boxed in (**C**,**D**), respectively. (**E**–**G**) H&E (E14.5) images show well developed interventricular septa in the control (**E**) but CKOs show atrioventricular septal defects, perimembranous ventricular septal defect, and abnormal ventricular myocardium (**F**,**G**). Additionally, AV cushions are dysmorphic and abnormally remodeled, compared to littermate/age-matched wild type embryos. (**H**,**I**) Although the surface area of AV cushions (**H**) shows a decreasing trend in CKOs, the data are not statistically significant (*p* > 0.05, Student’s *t*-test). There is significant thinning of the right ventricular myocardium (*p* = 0.011, two-tailed Student’s *t* test with Welch’s correction; *n* = 4) (**I**). Scale bars, 200 µm (**A**–**D**,**E**–**G**); 100 µm (**C′**,**D′**).

Then, we lineage traced cardiomyocytes over the course of embryonic development using *mTmG^+/−^* Cre-dependent reporter [26]. *cTnt*Cre robustly marks cardiomyocytes in the atria, ventricle as well as the septum separating the right and the left ventricular chambers (Figure 1E,H). In addition, there were few *cTnT*Cre-expressing cushion cells which did not express a myocardial marker (Figure S3). The atrioventricular (AV) cushions were found to be significantly myocardialized in some CKOs at E14.5, while all CKOs exhibited dysmorphic cushions and AV septation defects (Figure 1E–G and Figure 2E–I). The ventricular septal defect (VSD) in the CKOs seen at mid-gestation is due to the AV cushion remodeling defects and persist in the CKOs until later stages of development (E17.5; Figure 1H–J).

### 3.2. Conditional Deletion of Tgfb2 in Early Cardiomyocytes Leads to Cushion Remodeling Defect, Severe Thinning of Right Ventricle, and Muscular Type VSD

Histological analysis using H&E and cardiac muscle actin immunostaining showed normal aortopulmonary septation in CKOs, however, there were a few cases of OFT cushion thickening (Figure 2C,D and Figure S4A,B). Additionally, about 80% cases of CKOs developed interventricular septal defects including perimembranous, atrioventricular, and muscular VSDs (Figure 2A,B,E–G and Figure S4C,D). The development of the right ventricular (RV) myocardium, which is distinctly originated from the second-heart field, was particularly affected in the absence of cardiomyocyte-produced TGFβ2. We found myocardial thinning, particularly in the RV, as a highly penetrant phenotype in the CKOs (Figure 2C–D′,I and Figure S4E,F). Such a severe loss of compaction in the right, but not the left, ventricular myocardium (such as in Ebstein anomaly) led to thin compact myocardium (fewer layers) with prominent elongated trabecula (myocardial non-compaction). The cardiac malformations in response to the myocardial deletion of *Tgfb2* are summarized in Table 1.

CKOs had dysmorphic AV cushions compared to littermate controls (Figure 2E–G). We used morphometric measurements from H&E-stained serial sections to calculate the area of AV cushions in E12.5–14.5 embryos. Cushion size is known to reduce as it matures and contributes to AV valves. Thus, we compared cushion size separately in two groups E12.5–13.5 and E14.5. AV cushions showed a trend towards a smaller size in CKOs compared to wild-type littermates in both groups (average area (in μm^2^) E12.5–13.5: CKO = 3.03, Ctrl = 3.60; E14.5: CKO = 2.29, Ctrl = 2.96; *n* = 3–4, *p* > 0.05 in each group). To account for the spread in each group, we compared the median values which is higher in controls compared to the CKO in both age groups (Figure 2H). *Tgfb2* germline knockout embryos have also been reported to have increased apoptosis in AV valve leaflets due to a one-day lag in peak apoptotic activity compared to wild-type embryos. Interestingly, both the mean and median cushion area are comparable between E12.5–13.5 CKOs and E14.5 controls (3.03 vs. 2.96), suggesting a developmental delay in cushion maturation in the *Tgfb2* conditional mutants.

### 3.3. Cardiomyocyte-Derived TGFβ2 Is Required for Cushion Remodeling during Heart Development

**Figure 3:**
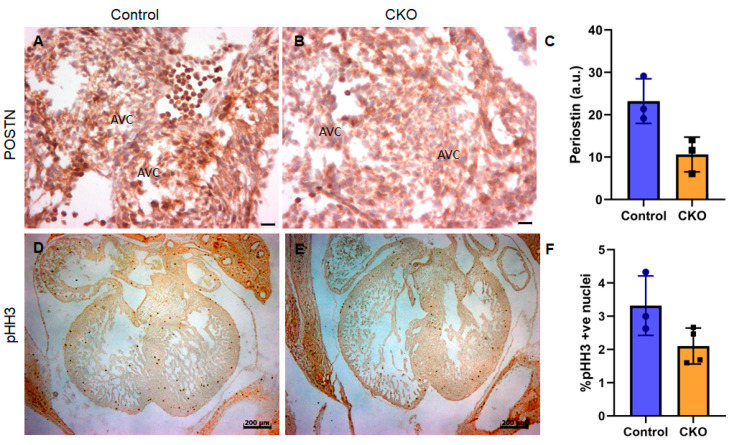
Cardiomyocyte-specific loss of *Tgfb2* results in defective cushion remodeling and myocardial cell proliferation. (**A**–**C**) Periostin immunohistochemistry. Myocardial *Tgfb2* CKOs have significantly reduced periostin expression (Mann–Whitney test, *p* < 0.05) in their endocardial cushions, compared to littermate controls (**D**–**F**). Phospho-histoneH3 immunohistochemistry and the quantification of myocardial cell proliferation between the two groups at E12.5–14.5 (Mann–Whitney, Exact *p* = 0.0571 (median: ctrl 3.0, cko 2.08)). Scale bars, 20 μm (**A**,**B**); 200 µm (**D**,**E**).

To investigate cushion dysmorphism in the CKOs, we used periostin, which is an established marker for cushion maturation and differentiation [27]. Its expression was significantly reduced in the CKO cushions (E12.5–13.5) compared to wild-type littermates (Figure 3A–C), suggesting impairment in cushion maturation in the absence of TGFβ2. Since TGFβ2 has established mitogenic properties, we hypothesized that cell proliferation would be reduced in the myocardium of CKOs. However, phospho-histone H3 (pHH3) immunohistochemistry revealed proliferation to be only slightly decreased in the CKOs (mean %pHH3 positive nuclei = 2.62, *n* = 3) compared to littermate controls (2.75, *n* = 3) and although there was a trend towards downregulation, the difference was not statistically significant (*p* = 0.057) (Figure 3D–F). Cell death (TUNEL) analysis showed that cushion apoptosis is also reduced in the CKOs, although the extent of reduction was greater in the outflow tract cushion, compared to AV cushions (Figure S5).

### 3.4. TGFβ Signaling Is “Paradoxically” Increased upon Myocardial Tgfb2 Deletion

**Figure 4:**
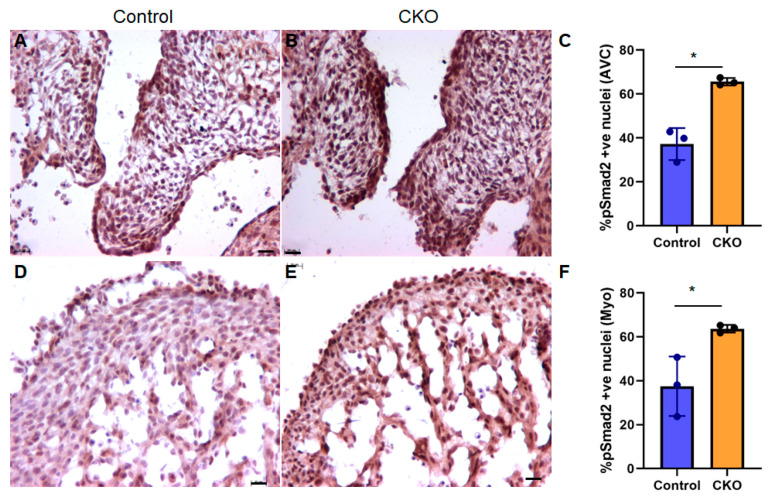
SMAD2 activation is increased in myocardial *Tgfb2* CKO hearts. (**A**–**F**) phospho-SMAD2 immunohistochemistry shows that CKOs have higher pSMAD2 (i.e., surrogate marker of TGFβ signaling) in both AV cushions (**A**–**C**) and myocardium (**D**–**F**), compared to littermate wild type controls at E13.5–14.5 (AV cushions: unpaired *t*-test with Welch’s correction, two-tailed, * *p* = 0.0172 (mean: ctrl 37.2, cko 65.61); Myocardium: unpaired *t*-test, two-tailed, * *p* = 0.029 (mean: ctrl 37.5, cko 63.7). Asterisks indicate statistically significant values (**C**,**D**). Scale bars, 20 μm.

Since the myocardial deletion of *Tgfb2* affects cushion remodeling, a structure that is not derived from cardiomyocytes, we reasoned that it might represent a non-cell autonomous effect as TGFβs are secreted out. To circumvent this, we looked at TGFβ signaling via canonical receptor SMAD2 which is known to be phosphorylated upon activation and being an intracellular signal transducer, it acts in a cell autonomous fashion. The level of phosphorylated SMAD2 (p-SMAD2) acts as a surrogate for the signaling activity. The loss of TGFβ2 is expected to decrease pSMAD2. Instead, we found that p-SMAD2 expression in both AV cushion (Figure 4A–C) and ventricular myocardium (Figure 4D–F) was “paradoxically” increased, compared to wild-type littermate controls at E13.5–14.5 (*p* < 0.05, for both cushion and myocardium, respectively; Mann–Whitney test). Interestingly, the density of nuclei expressing phosphorylated SMAD2 directly correlates with the phenotypic defects, especially myocardial thinning, and cushion dysmorphism, in the CKO embryos (embryo with the weakest cardiac defects has the lowest %pSMAD2-positive nuclei, among the many CKOs analyzed). Although the range of values is wider for the wild-type than for CKOs (Figure 4C,F), we obtained similar levels of pSMAD2 signal in both cushion and myocardium for an individual animal. Collectively, our data indicated that the loss of myocardial TGFβ2 results in an unexpected elevated TGFβ signaling via SMAD2 in both AV cushions and myocardium, suggesting both an autocrine and paracrine role of myocardial TGFβ2 in heart development.

## 4. Discussion

TGFβs are a superfamily of multifunctional cytokines involved in development through regulating processes such as cell division, differentiation and migration, epithelial-to-mesenchymal transition, extracellular (ECM) matrix expression and ECM remodeling [1]. The complexity of TGFβ signaling is in its context dependency, built-in feedback or compensatory mechanisms, and crosstalk with other signaling cascades [28,29]. Among the three TGFβ ligands, *TGFB2* mutations which cause Loeys–Dietz syndrome 4 (LDS type 4) have been linked to aortic aneurysm and rupture and congenital heart defects, including valve malformations and septation abnormalities [8,30,31]. *TGFB2* signaling has also been involved in cardiac myxoma tumor histogenesis [32]. *Tgfb2* null fetuses die perinatally and exhibit a spectrum of cardiac malformations including double outlet right ventricle, common arterial trunk, abnormal cushions, thick semilunar and AV valves, as well as AV septal defects (AVSD) [8,12,13,20,22]. TGFβ2 promotes myogenesis and *Tgfb2* null mice exhibit the defective myocardialization of AV cushion mesenchymal complex and spongy myocardium. *Tgfb2* is expressed right from the pre-cardiac mesoderm stage and produced by multiple cell types, corroborating its pivotal role in mammalian heart development [33]. However, the cell lineage specific functions vis-à-vis autocrine–paracrine roles of TGFβ2 in heart development are currently unknown. Our lab has previously generated *Tgfb2^flox^* mice which allow to conditionally delete genes in specific cell types in order to determine the function of *Tgfb2* in a spatio–temporal fashion [20]. The current study looks at the role of TGFβ2 produced by cardiomyocytes, one of the major cell types in the mammalian heart. *Tgfb2* is highly expressed by the OFT and ventricular myocardium in early heart, suggesting its critical role in OFT and chamber morphogenesis [5,6]. To decipher the role of myocardial TGFβ2 in cardiac development, we deleted *Tgfb2* from cardiomyocytes using the *cTnt*Cre which specifically and effectively deletes *Tgfb2* in cardiomyocytes, reflected in reduced expression in the ventricular myocardium [24]. Our data suggest that myocardial TGFβ2 is important for ventricular development (especially RV) including the compaction and trabeculation of ventricular myocardium. Although myocardial TGFβ2 is somewhat required for OFT cushion remodeling, it seems dispensable for OFT morphogenesis, alignment and septation. While the myocardial phenotype is anticipated, the effect on AV cushion remodeling—dysmorphic, smaller AV valve—in *Tgfb2* myocardial CKOs indicates a non-cell autonomous or paracrine role of the TGFβ2 ligand that is produced by AV myocardium and secreted out and acts on AV cushion mesenchyme. Such instances of TGFβ ligand produced by one cell acting on another have previously been documented [34]. Although an indirect role of TGFβ2-produced by AV myocardium in AV cushion remodeling and AV septation has been suggested previously by others [35], there has been no direct demonstration of endocardial cell lineage-specific TGFβ2 function in AV cushion remodeling and AV septation. Ongoing experiments in the laboratory will reveal the role of endocardial TGFβ2 in heart development.

Given the importance of TGFβ2 myocardialization [23], this study provided direct evidence of a role of myocardial TGFβ2 in the muscularization of the AV septum. Since *Tgfb2* myocardial CKO embryos exhibit AV cushion fusion and septal abnormalities, it is difficult to determine a precise role of myocardial TGFβ2 in the myocardialization of AV cushions. Although apoptosis in the AV cushion mesenchymal complex was significantly reduced, the myocardialization of the AV cushions is not significantly affected in *Tgfb2* myocardial CKO embryos. On the contrary, the cell lineage tracing of myocardial cells in some *Tgfb2* myocardial CKO embryos unexpectedly indicate the abnormal accumulation of myocardium in AV mesenchymal complex. Since second heart field (SHF)-derived cells also contribute to AV cushion complex [36], it is plausible that a lack of myocardial TGFβ2 in the AV myocardium allows the increased recruitment and differentiation of SHF-derived cells. The increased myocardialization of AV cushions in some mutants could be interpreted as secondary to the increased SMAD2 activation, which is known to have myogenic differentiation and commitment [37]. SHF-derived cells (endocardial and/or myocardial cells) also contribute to RV [36,38]. Myocardial thinning is particularly severe in the right ventricle (RV) of *Tgfb2* myocardial CKOs. There were no myocardial defects in embryos with a lack of myocardial TGFβR1 or TGFβR2 [39,40]. Thus, a loss of myocardial TGFβ2 acting in a paracrine fashion on non-myocardial cells (e.g., SHF-derived cells) is required for proper myocardial proliferation and myocardial development. Further investigation is needed to determine if *Tgfb2* is required for the development of the SHF-derived OFT and right ventricular myocardium and SHF-derived structures of the AV mesenchymal complex (i.e., dorsal mesenchymal protrusion).

Our study contradicts previous reports documenting TGFβ signaling to be redundant in the myocardium. The myocardial deletion of *Tgfbr2*, the receptor of an integral part of the complex which transduces TGFβ2 signal, rarely leads to any cardiac defects in embryos [40]. Interestingly, *Tie2Cre* mediated the endocardial deletion of *Tgfbr2* which specifically affects the inferior AV cushion mesenchyme and leads to reduced mitosis (and double inlet left ventricle) and produces smaller, dysmorphic cushions; and muscular VSD, as reported in the current study. Cell lineage-specific deletion of *Tgfbr1* also failed to indicate any significant cell autonomous role of TGFβ signaling in myocardium during heart development [39]. Since TGFβ receptors function autonomously from cells and TGFβ2 can act both in autocrine and paracrine fashion, our results indicate that myocardial TGFβ2 acts on endocardial cell lineage (endocardium and/or endocardial-derived AV cushion mesenchyme) to facilitate their proper myocardial–endocardial cell interactions involved in myocardial development and AV cushion remodeling and AV septation. One of the limitations in this study was the specificity of the rat c*TnT* or *TnnT2* promoter for cardiomyocytes, where the use of *Tnnt2Cre* mice results in the labeling of intercalated cushion cells [41,42]. These cushion cells retain signatures of previous activation of the cardiac troponin promoter, although they do not have a myocardial phenotype. These cushion cells are not derived from differentiated myocardial cells but arise instead from the direct differentiation of SHF progenitors into valve cells. Future investigation should delineate the role of TGFβ2 in myocardium using another *Cre* driver such as *Nkx2.5Cre*, and endocardial cell lineage in heart development, which should further reinforce the cell-specific requirement of TGFβ2 in cardiac morphogenesis.

Importantly, we have found a ‘paradoxical’ increase in TGFβ signaling in both AV cushions as well as the ventricular myocardium in CKO hearts. Such an increase in TGFβ signaling upon knocking out one TGFβ ligand is not uncommon, as reported by us and others [34,43]. The data presented here suggest that the loss of *Tgfb2* from cardiomyocytes may activate a compensatory mechanism to restore signaling through a feedback mechanism which likely involves other TGFβs or increased TGFβ receptors expression. This might represent a feedback mechanism to restore homeostasis, an idea supported by the observation that the CKO with some residual *Tgfb2* expression also has the lowest percentage of pSMAD2-positive nuclei within the group and displays a weaker cardiac phenotype. The reduced AV cushion size in the CKOs suggests that AV cushion mesenchymal cells, which normally respond to myocardial TGFβ2, probably have a proliferation defect due to cell cycle arrest in the absence of this mitogenic signal. This speculation is consistent with reduced cell proliferation and cyclin D1 levels in AV cushion cells of endocardial cell lineage *Tgfbr2* knockout embryos which develop AV cushion remodeling defect and incomplete AV septation [40]. It has been reported that a “paradoxical” increase in TGFβ signaling contributes to mitral valve disease in the Marfan syndrome mouse model [44]. mitral valve prolapse (MVP) with mitral regurgitation has been observed in individuals with *TGFB2* mutations (such as Loeys–Dietz Syndrome), although less frequently than in the Marfan syndrome [45]. Thus, the results of this study have significant implications in understanding the developmental basis and etiology of MVP in LDS. In conclusion, myocardial TGFβ2 plays an essential role in myocardial development and AV cushion remodeling and AV septation during heart development.

## Figures and Tables

**Table 1:** Summary of cardiovascular defects in *cTnt*Cre*; Tgfb2*^−/f^ E12.5–18.5 embryos (*n* = 13 CKOs).

ID	Age	Cardiac Defects			
		OFT Cushion Thickening	VSD	AV Cushion and Septation Defects	Myocardial Defects
CKO-1	E12.5–13	ND	Yes	Slightly smaller but not dysmorphic, incomplete AVSD	Yes; RV more affected than LV,
CKO-2	E12.5–13	ND	Yes	Smaller, dysmorphic, incomplete AVSD	Yes; RV more affected than LV,
CKO-3	E12.5–13	ND	Yes	Dysmorphic, incomplete AVSD	yes
CKO-4	E12.5	ND	ND	ND	
CKO-5	E13.5	Yes	ND	ND	Yes; RV more affected than LV
CKO-6	E14.5	yes	Muscular	Smaller but not dysmorphic	Yes
CKO-7	E14.5	ND	Yes	Dysmorphic, incomplete AVSD	Yes
CKO-8	E14.5	ND	Yes	Dysmorphic, incomplete AVSD	Yes
CKO-9	E16.5	No	No	AV valves normal	Yes
CKO-10	E16.5	No	Muscular	AV valves normal	Yes; RV more affected than LV
CKO-11	E17.5	No	Yes	ND	Yes
CKO-12	E17.5	ND	Perimembranous	ND	Yes
CKO-13	E18.5	Yes	No	ND	Yes
Total CKO	13				
Controls	19				

CKO, conditional knock-out; ND, not determined; OFT, outflow tract; AV, atrioventricular; VSD, ventricular septal defect; AVSD, atrioventricular septal defect; RV, right ventricle; LV, left ventricle.

## Data Availability

The data presented in this study are available in supplementary material here. The raw data of this study are available from the corresponding author upon reasonable request. Correspondence and requests for materials should be addressed to M.A.

## Supplementary Materials

The following are available online https://www.mdpi.com/2308-3425/8/3/26/s1, Figure S1. A schematic of the genetic cross to produce myocardial *Tgfb2* CKO embryos and the details of the experiments performed over the course of embryonic development; Figure S2. CKOs (*Tgfb2**^+^*^/flox^; *cTnt*Cre^Tg^; *Tgfb2*^+/−^) (B,D) are slightly smaller than the littermate controls (A,C), but are otherwise grossly normal (A,B E9.5, C,D E13.5). Scale bars, 500 µm (A–D); Figure S3. Efficiency of Cre recombination. *cTnt*Cre expression marked by GFP fluorescence (A, green) of *mTmG^+/−^* reporter allele is completely overlapped with a myocardial specific marker, cardiac α-actin (B, red) in the entire E13.5 control heart (D). All nuclei are marked with DAPI (C, blue). There are some GFP-expressing OFT cushion cells that do not express myocardial marker, which is consistent with the published literature (see Discussion). Scale bars, 100 µm (A–D); Figure S4. (A,B) Conditional deletion of *Tgfb2* in early cardiomyocytes causes outflow tract cushion (oftc) thickening. Cardiac muscle actin staining by MF20 IHC shows normal OFT of the littermate wild-type control but OFT in myocardial *Tgfb2*CKO (E13.5) embryo is thickened. (C,D) H&E staining showing defective development of interventricular muscular septum (IVS) in (E13.5), resulting in muscular ventricular septal defect (VSD). (E, F) Myocardial *Tgfb2* CKO (E18.5) embryo have thin compact as well as trabecular myocardium in the right ventricle compared to a control littermate fetus. Scale bars, 100 μm (A–F); Figure S5. Apoptosis. (A–E) TUNEL showing reduced apoptosis in outflow tract (OFT; A–B) and atrioventricular cushions (AVC; C–D) in CKOs compared to littermate control embryos. A’–D’ and C’–D’ represent magnified views of A–D and C,D, respectively. E. CKOs have a significantly smaller number of TUNEL positive (i.e., apoptotic) nuclei in the endocardial cushions (*p* = 0.021, unpaired *t*-test with Welch correction) than the littermate controls (*n* = 3 for each group). Scale bars, 200 µm (A,B,C,D); 100 µm (A′,B′,C′,D′).
