# Supplementary figures and images for: Myocardial TGFβ2 Is Required for Atrioventricular Cushion Remodeling and Myocardial Development
Source: J Cardiovasc Dev Dis. 2021 Mar 2;8(3):26. doi: 10.3390/jcdd8030026 (PMC7999251; doi:10.3390/jcdd8030026)

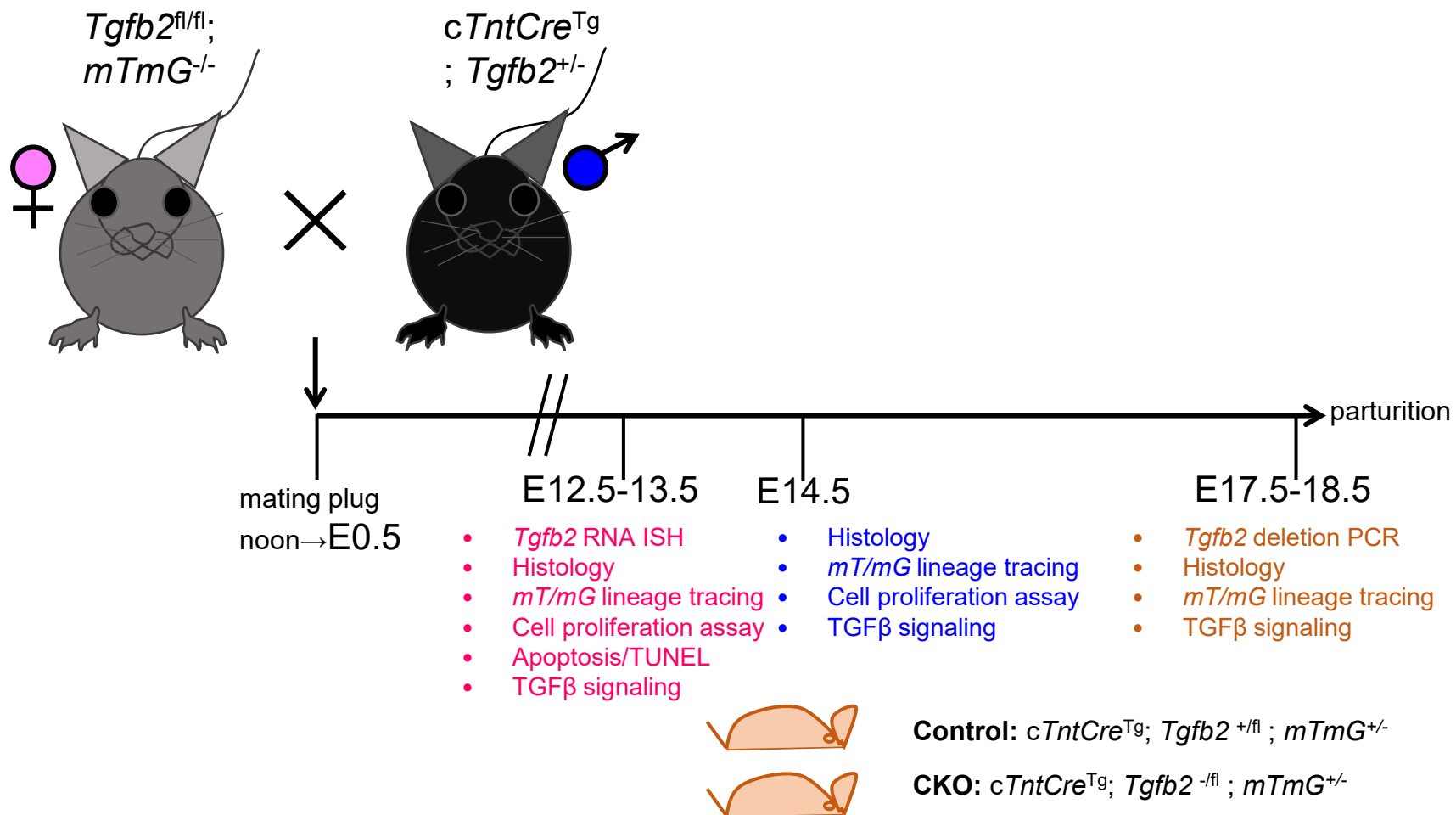

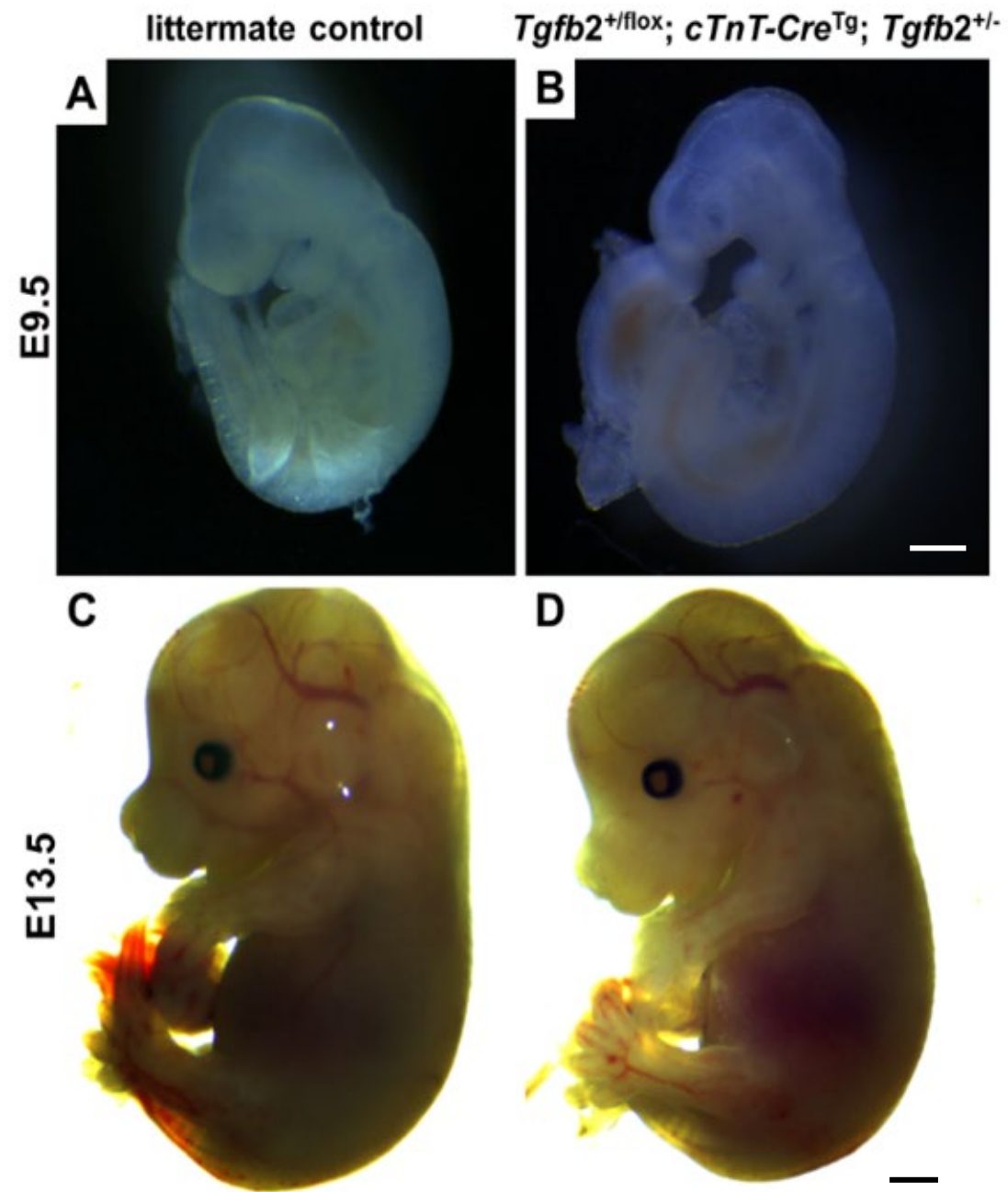

Combined

DAPI

Cardiac actin

GFP

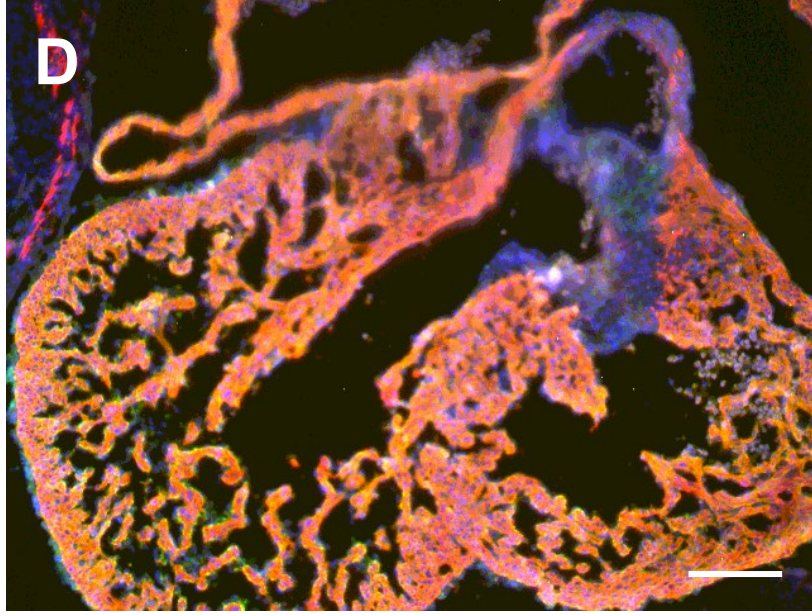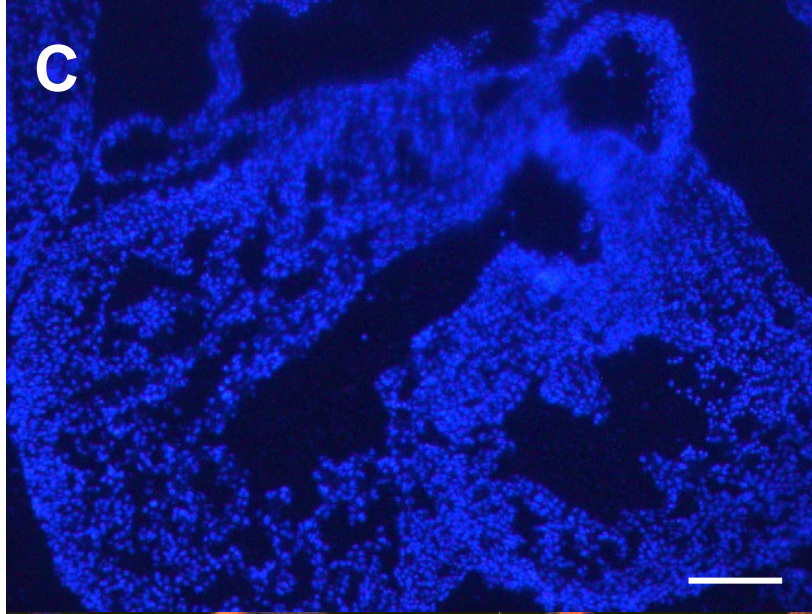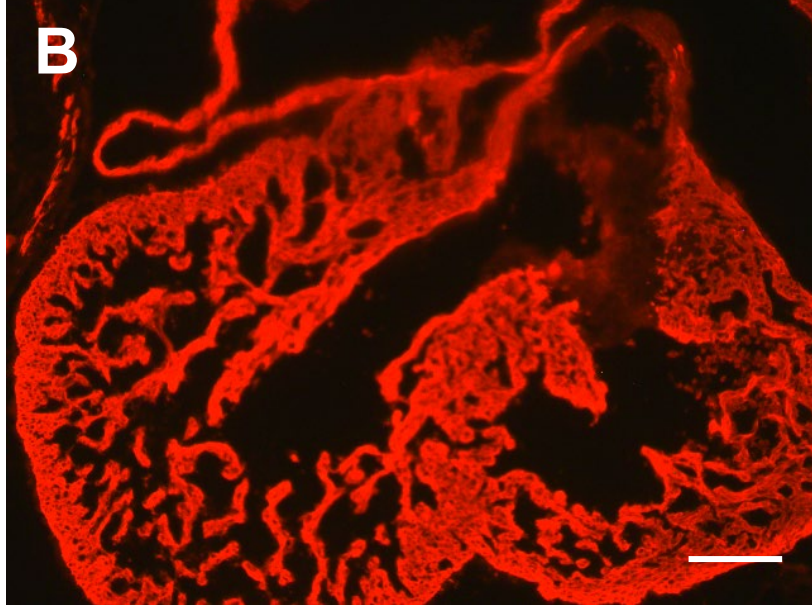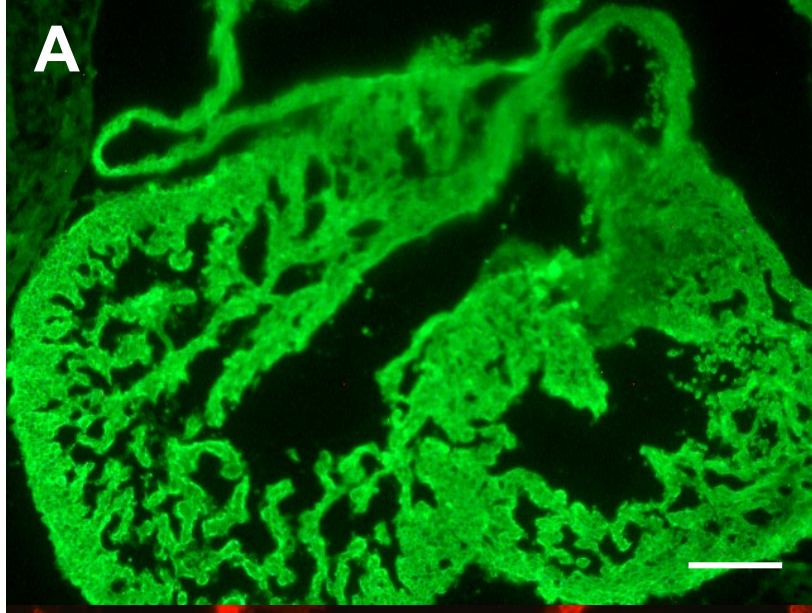

Control

CKO

E13.5

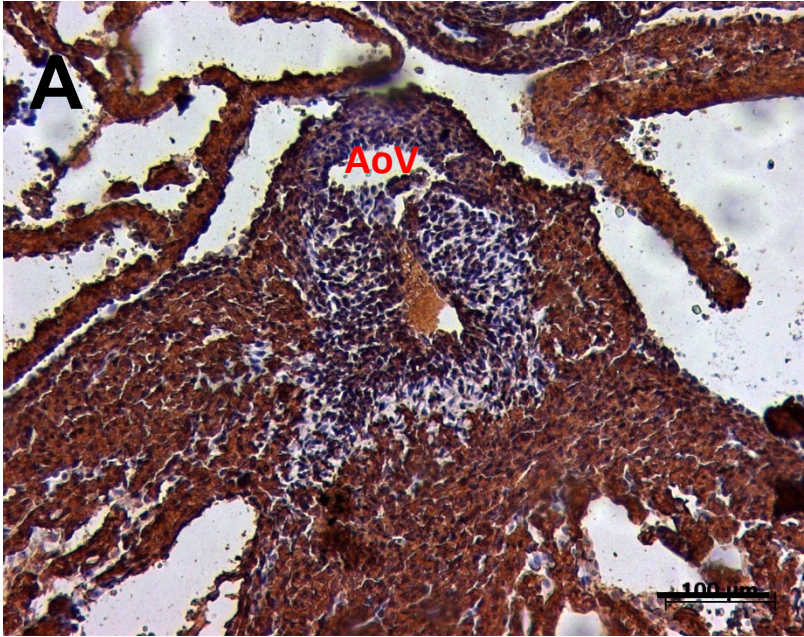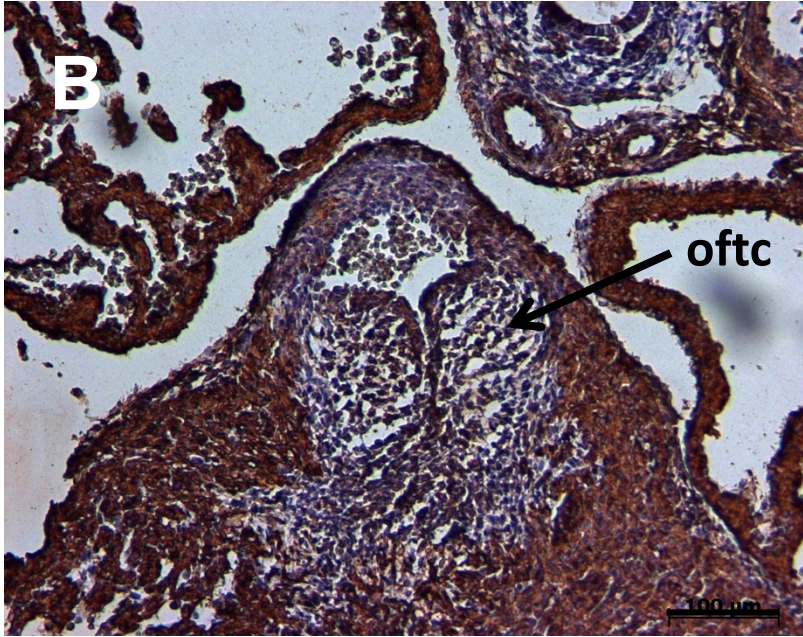

E13.5

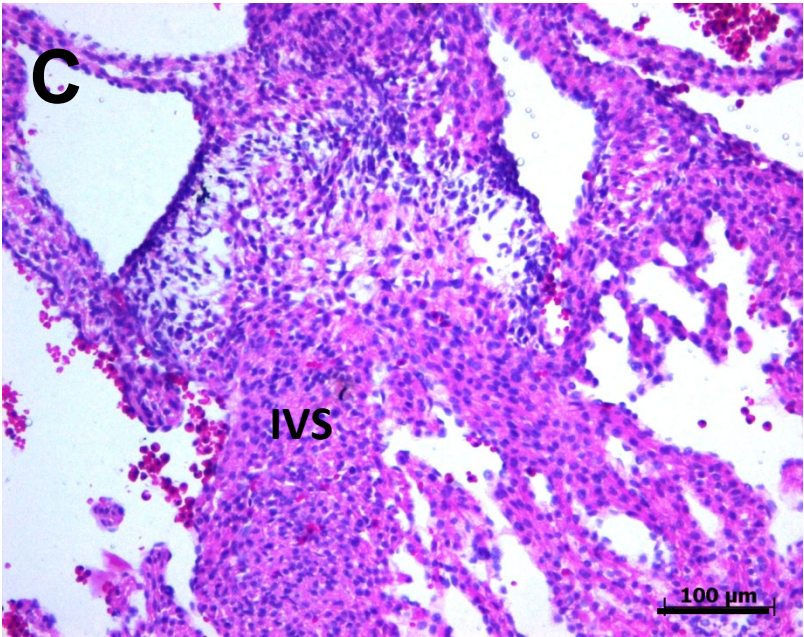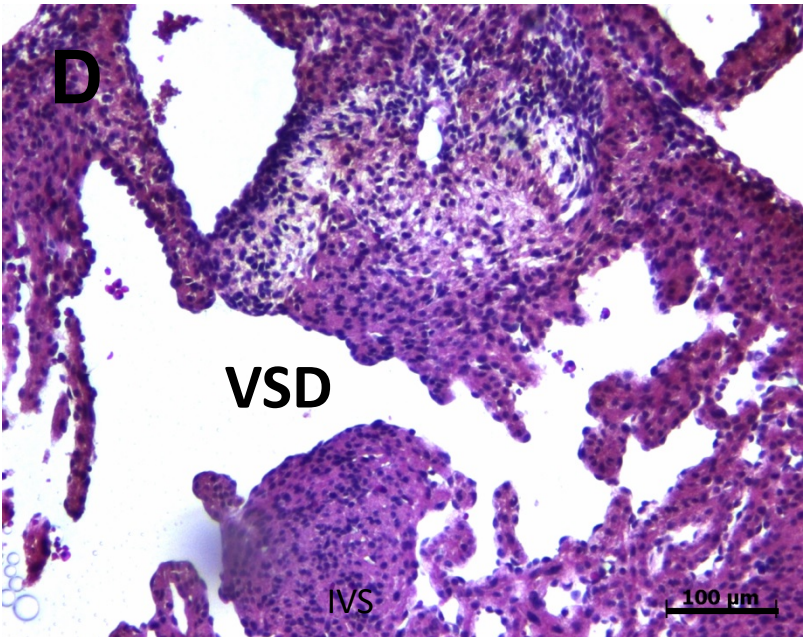

E18.5

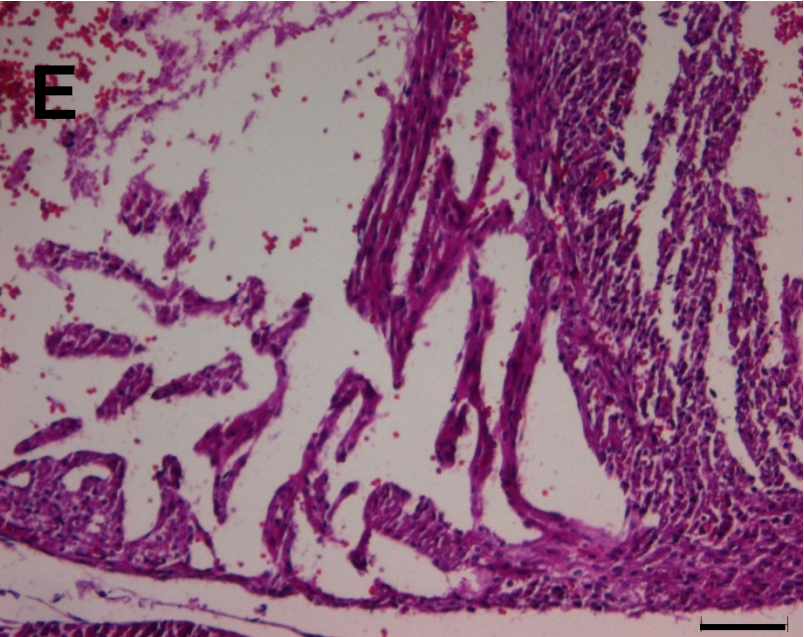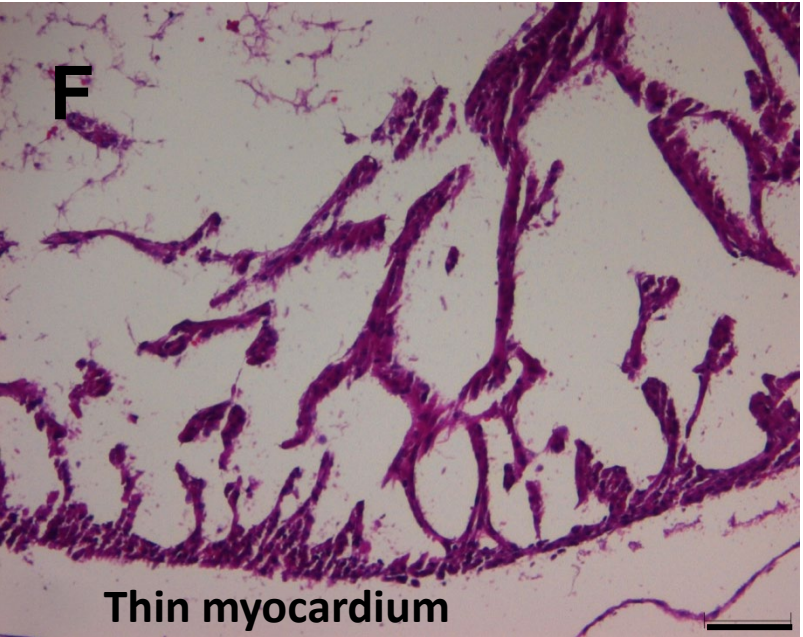

**Control**

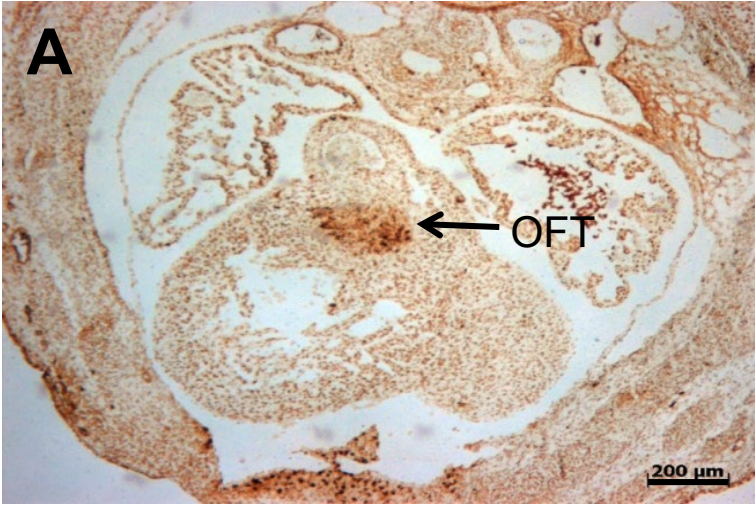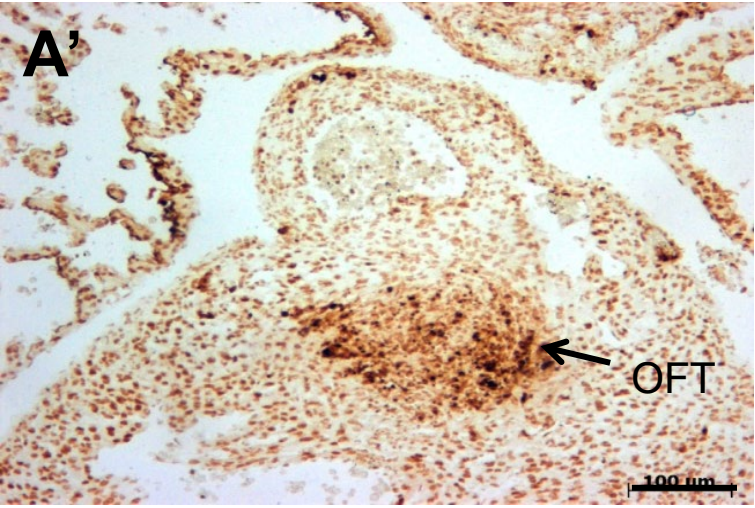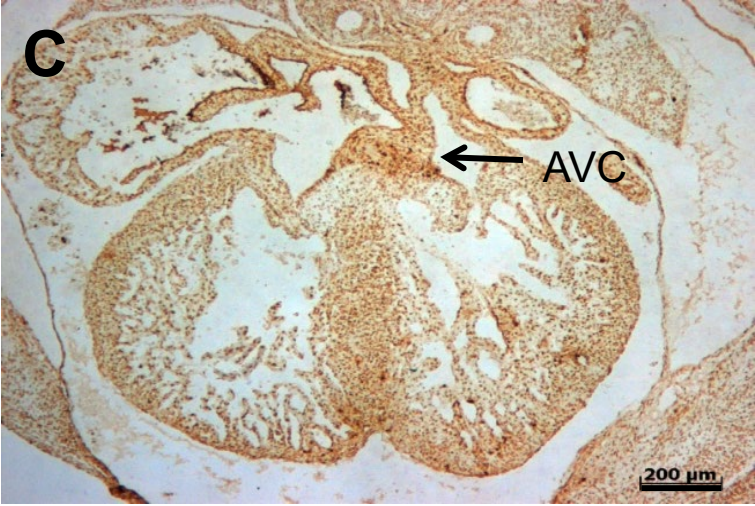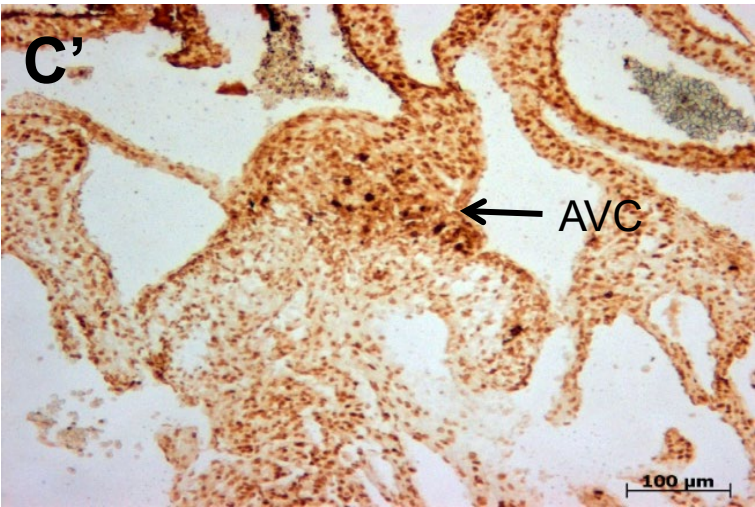

**CKO**

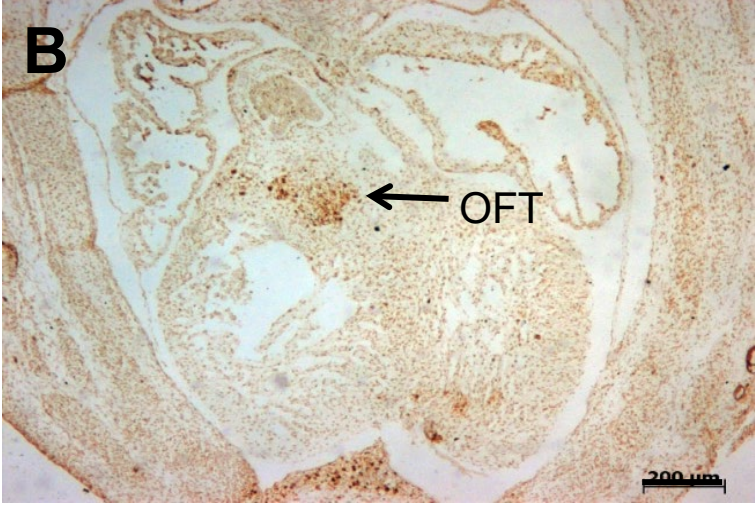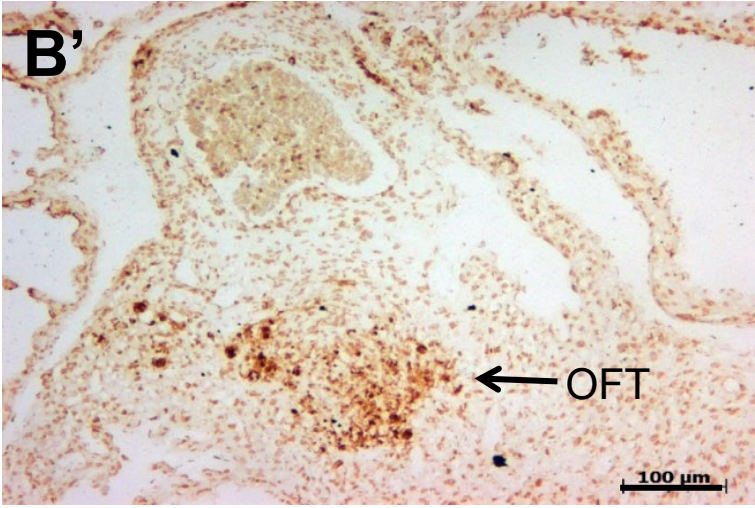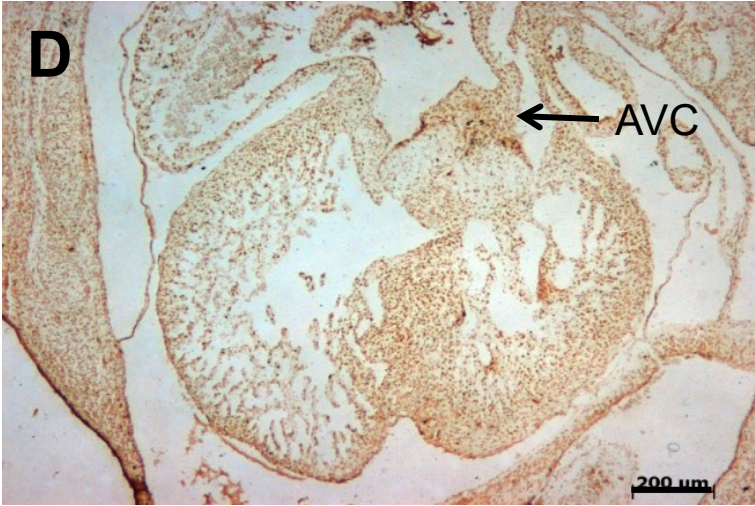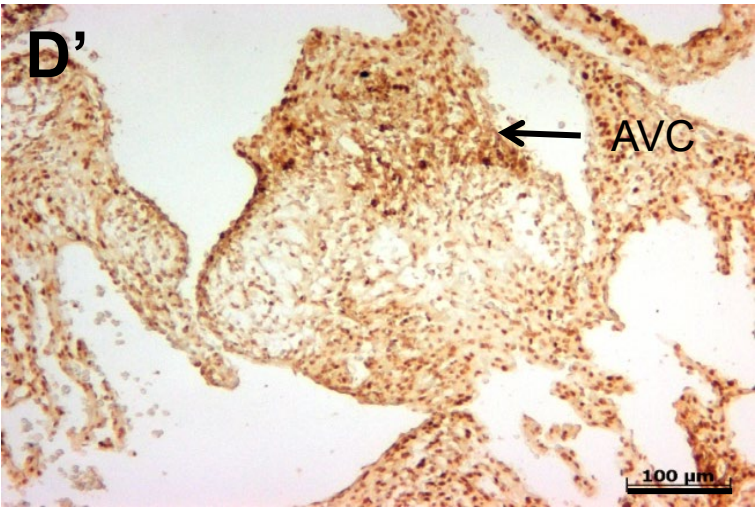

**E**

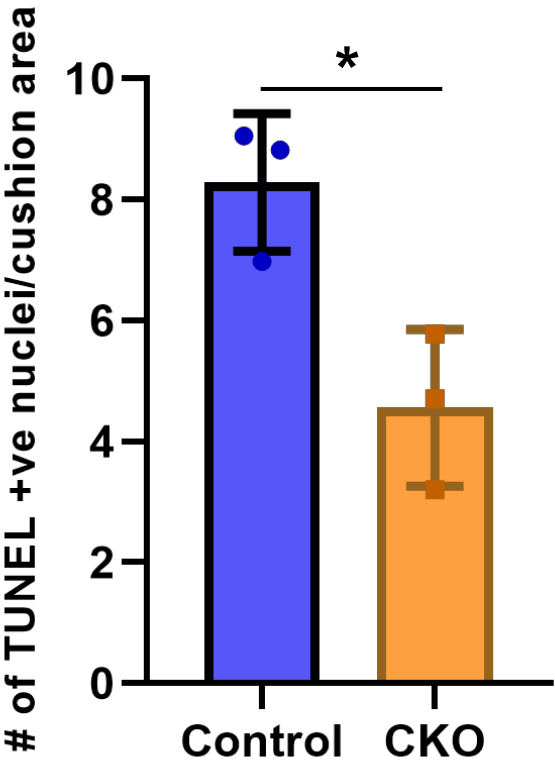

Supplement: Supplementary file 1 [file jcdd-08-00026-s001.pdf]
